# Detecting potential causal relationship between inflammatory bowel disease and rosacea using bi-directional Mendelian randomization

**DOI:** 10.1038/s41598-023-42073-6

**Published:** 2023-09-09

**Authors:** Min Li, Si Xian He, Yuan Xiong He, Xiao Han Hu, Zhou Zhou

**Affiliations:** Department of Dermatology, People’s Liberation Army The General Hospital of Western Theater Command, Chengdu, China

**Keywords:** Gastrointestinal diseases, Skin diseases, Computational biology and bioinformatics, Genetics, Medical research

## Abstract

The association between rosacea and inflammatory bowel disease (IBD) has been studied in previous observational studies. It is unclear, however, whether the association is causal or not. Independent genetic variants for IBD were chosen as instruments from published Genome-wide association studies (GWAS) studies involving 38,155 cases with an IBD diagnosis and 48,485 controls in order to investigate the causal effect of IBD on rosacea. Summarized data for rosacea were gathered from various GWAS studies that included 1195 cases and 211,139 controls without rosacea. Reverse-direction Mendelian randomization (MR) analysis was done to investigate the relationship between genetically proxied rosacea and IBD. With the use of the inverse variance-weighted (IVW), MR-Egger, and weighted median approaches, a 2-sample Mendelian randomization study was carried out. Analysis of heterogeneity and sensitivity was performed to examine the pleiotropy and robustness of effect estimates. The forward-direction of the MR study was to reveal that genetic predisposition to IBD including its two main subtypes: Crohn’s disease (CD) and ulcerative colitis (UC) was associated with an increased risk of rosacea. The reverse-direction MR analyses did not demonstrate that a genetic predisposition to rosacea was associated with total IBD, UC and CD. Our findings provided evidence for a causal impact of IBD, UC, and CD on rosacea, but not vice versa. The elevated incidence of rosacea in patients with IBD should be recognized by doctors to make an early diagnosis and initiate specialized therapy.

## Introduction

Rosacea is a chronic inflammatory skin condition that produces erythema of the central face region that can be transitory or permanent^1^. The prevalence of rosacea was estimated at 5.46% in adults, especially those between ages 45 and 60^2^. Rosacea can negatively impact self-esteem, confidence, and quality of life in the case of persistent facial erythema^3^. Rosacea has been linked to cardiometabolic illness, *Helicobacter pylori* infection, Demodex infection, inflammatory bowel disease, migraine, depression, and anxiety in several studies^3–9^ Inflammatory bowel disease (IBD) including Crohnness, *Helicobacter pylori* infection, Demodex infection, inflammatory bowel disease, migraine, depression, and anxiety in several studiesb-id = "0wf9te59brx9xport an underlying bidirectional relationship between rosacea and IBD, although mechanisms remain unclear^8,10–13^. Many common genetic, environmental, and pathophysiologic factors are shared by rosacea and IBD^14^. The rs763035 is a significant single-nucleotide polymorphism (SNP) associated with rosacea^15^. rs763035 is intergenic between HLA-DRA, a major histocompatibility complex class I allele, and butyrophilin-like 2 (BTNL2), while a study suggests that BTNL2 has been implicated in IBD^16^. Both smoking and alcohol consumption have been found to have a potential association with the presence of rosacea and IBD, according to a comprehensive review of multiple studies^17–20^. Both rosacea and IBD are linked with abnormalities in innate and adaptive immunity^14^. Inflammation in rosacea and IBD is mediated by activation of macrophages and toll-like receptor 2, dysregulation of mast cells, fibroblasts, and generation of reactive oxygen species, matrix metalloproteinases, tumor necrosis factor, and interleukin-1^14,21^. Regarding adaptive immunity, T helper type 1 and 17 cells, as well as B cells, are pathogenic in both rosacea and IBD due to the production of interferon-gamma, tumor necrosis factor, interleukin-17, and several immunoglobulins^14,21,22^.

Nevertheless, basic and observational studies are susceptible to confounding factors like demographics or environmental exposure including age, gender, smoking, alcohol consumption, and body mass index (BMI)^14,23–25^. Hence, rosacea and IBD remain controversial in terms of any causal bidirectional role.

The causal relationship between exposure and outcome can be assessed by using Mendelian randomization (MR) with genetic variations as instrumental variables^26^. This approach simulates the randomization process of a randomized controlled experiment by exploiting the random distribution of genetic variation to eliminate confounding factors and reverse causality^26^. In the present study, MR was used to evaluate the causal relationship between rosacea and IBD, along with its strength and direction of causality.

## Materials and methods

**Figure 1:**
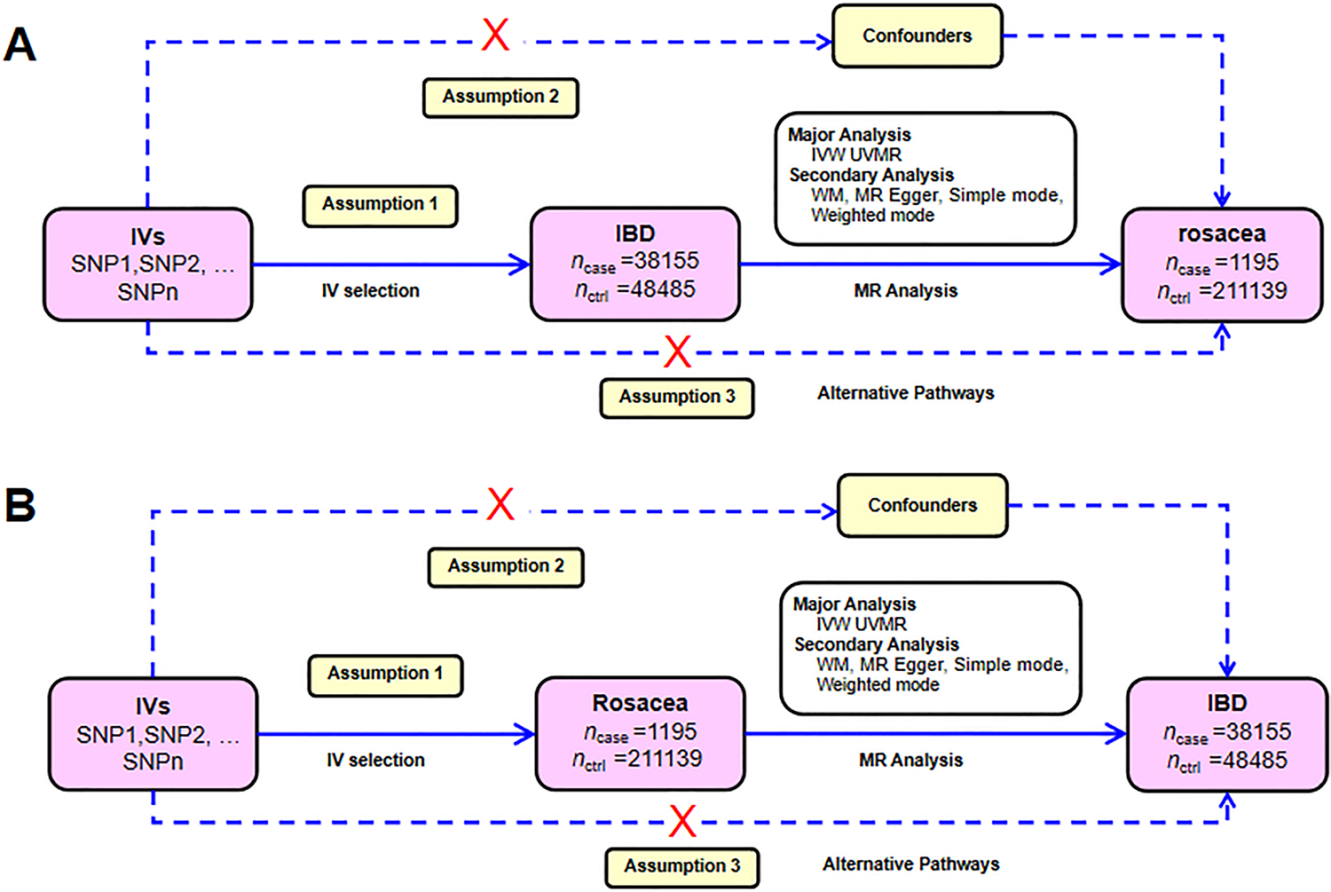
Diagram illustrating the main MR analysis assumptions. (**A**) IBD SNPs were employed as the genetic instruments to examine the causal effect of IBD on rosacea. (**B**) Rosacea SNPs were employed as the genetic instruments to examine the causal effect of rosacea upon IBD. *IBD* inflammatory bowel disease, *IVW* inverse variance-weighted, *MR* Mendelian randomization, *PRESSO* Pleiotropy RESidual Sum and Outlier, *SNP* single-nucleotide polymorphism, *UVMR* univariable Mendelian randomization, *WM* weighted median.

Multiple single-nucleotide polymorphisms (SNPs) representing genetic variation were selected as instrumental variables and analyzed using two-sample MR. The following three hypotheses were adopted (Fig. 1): (1) Instrumental variables (IVs) are directly and robustly related to exposure; (2) IVs are independent of any confounding factors; (3) IVs only affected outcomes via exposure^27^. MR analysis was conducted to assess bidirectional causal relationships between IBD and rosacea.

### Data source

Summary statistics for rosacea were obtained from the FinnGen Consortium which contained 1195 cases and 211,139 individuals without rosacea (FinnGen Biobank Analysis Consortium; GWAS ID: finn-b-L12_ROSACEA; n = 212,334 (1195 cases and 211,139 controls); https://gwas.mrcieu.ac.uk/datasets/finn-b-L12_ROSACEA/). The FinnGen project was launched in 2017 with the aim of collecting biological samples from 500,000 participants in Finland over six years with the aim of improving health through genetic research. Summary statistics for IBD were obtained from The IIBDGC Consortium which contained a total sample size of 86,640 participants of predominantly European ancestry (cases/controls for IBD: 38,155/48,485; UC: m17647/47,179; CD: 20,550/41,642; https://www.ibdgenetics.org/#downloads)^28^.

The SNPs and corresponding summary statistics used in our analyses were selected from studies enrolling only individuals with European ancestry for both IBD and Rosacea to avoid population bias. Because all of the data utilized is already in the public domain, no further ethical approval was required.

A series of quality control steps were implemented to select eligible IVs. The IVs for IBD were selected from the SNPs associated with IBD at genome-wide significance (*p* < 5 × 10^–8^) in the dataset. The SNPs were clumped to achieve independent loci with a threshold linkage disequilibrium (LD) *r*^2^ = 0.001 and 10,000 kb distance^29^. Then, the effect estimates of the selected IVs from the rosacea dataset were extracted. The SNPs for rosacea were selected that were genome-wide significant (p < 5 × 10^−6^) and independently inherited (r^2^ < 0.001, distance > 10,000 kb) without LD from finn-b-L12_ROSACEA GWAS dataset. Then, the corresponding effect estimates of the selected IVs from the IBD GWAS dataset were extracted. In the context of IBD-Rosacea relationship, Rosacea-relevant traits such as cardiometabolic disease, *Helicobacter pylori*, Demodex infection, inflammatory bowel disease, migraine, depression, and anxiety^4–9^ and IBD-relevant traits such as *Helicobacter pylori* and obesity^30,31^ are most likely to be potential and major confounders. To fulfill the second MR assumption, the PhenoScannerV2 database was used to search for IVs and their proxied traits and the IVs arrogating these traits at a threshold of p < 5 × 10^8^ and r^2^ > 0.80 were removed^32,33^. To harmonize the IBD and Rosacea data, all palindromic SNPs with intermediate allele frequencies were removed from the selected instrumental SNPs above^34^. These rigorously selected SNPs were used as the final instruments for the MR analyses. Detailed information on IVs is shown in Supplementary Tables 1–5.

### Estimation of causal effect

After the list of SNPs according to the selection criteria above was determined, a forward MR analysis was conducted to estimate the overall effects of the selected SNPs for IBD on Rosacea using multiple methods, including inverse variance weighted (IVW), MR-Egger regression, weighted median (WM), simple mode and weighted mode. Since the results could be biased by horizontal pleiotropy of the IVs, the validity of the results was checked by comparing the effect estimates across the five MR methods.

The reverse-direction MR analysis was then conducted using the same MR methods as above. Effect estimates were reported in odds ratios (OR) with 95% confidential intervals (CI).

### Heterogeneity and sensitivity analysis

SNP heterogeneity was quantified using Cochran Q-statistics and I^2^-values^35^. "Leave-one-out" analysis was used to assess the robustness of MR estimates by eliminating a different SNP in each iteration to quantify the causal influence of outlying SNPs and to ensure that deleting SNPs did not affect the MR estimates^36^. Pleiotropy RESidualSum and Outlier (MR-PRESSO) analysis was applied to outliers of instrumental variables with pleiotropic effects^37^.

### Instrumental strength and statistical power

An estimation of variance was performed using the formula R^2^ = 2 × MAF × (1 − MAF) × (beta/SD)^2^, where MAF denotes the effect allele frequency and beta is the allele effect estimate of the genetic variant^38^. The F statistic was calculated using the formula F = [(N − k − 1)/k] × [R^2^/(1 − R^2^)], where k denotes the number of SNPs, N is the sample size, and R^2^ denotes the proportion of exposure variability explained by genotype^39^. The F-statistic > 10 indicates a sufficiently strong instrument to explain phenotypic variations, whereas the F-statistic ≤ 10 indicates a weak instrument^40^.

### Statistical analysis

Statistical analyses were performed using the TwoSampleMR (version 0.5.6) and MR-PRESSO (version 1.0) packages in R (version 4.2.1), and plots were created by the ggplot2 package. All statistical tests were performed on both sides, and the findings of the MR analysis and sensitivity analysis regarding exposures and outcomes were judged statistically significant at *p* < 0.05. The concept and processes for this MR study are illustrated in Fig. 1.

### Ethics statement

Ethical approval and informed consent were obtained from the original GWAS, no separate ethics statement was required for this study.

## Results

### The causal effect of IBD and its subtypes on Rosacea

To investigate the causative influence of IBD on Rosacea risk, significant at the genome-wide level (*p* < 5 × 10^−8^) and independently inherited (*r*^*2*^ < 0.001 and distance > 10,000 kb) from the 12,716,084 SNPs, 63 SNPs were tentatively selected as IVs for IBD. By enquiring about the PhenoScanner V2 database, three SNPs related with at least one Rosacea-relevant trait, such as rs6062496, were ruled out. 10 SNPs were not available in the summary-level datasets of Rosacea. 6 potential SNPs were found to be palindromic. Finally, 44 SNPs were approved for MR analysis of IBD's causative influence on rosacea risk. Similarly, according to the above processes and standards, 25 and 34 SNPs were screened as genetic instruments for UC and CD respectively. Based on F statistics and the proportion of variance explained (R^2^), all genetic instruments were suitable for MR analysis. Supplementary Tables 1–5 provide a summary and full descriptions of the IVs for each exposure.

Cochran's Q test revealed no significant heterogeneity in the analysis of total IBD on rosacea. (Q = 54.1571; *p* = 0.1184). Thus, fixed-effect models were applied in the IVW analysis. There was no directional pleiotropy bias found in the MR-Egger test (Intercept = 0.0018; *p* = 0.9408). No SNP outliers were found in the MR-PRESSO global test (RSSobs = 56.5382; *p* = 0.1400) and leave-one-out MR analysis. There was a strong association discovered between overall IBD and rosacea (IVW: OR 1.1291; 95% CI 1.0444–1.2206; p = 2.30E−03). WM (WM: OR 1.1455; 95% CI 1.0226–1.2832; p = 0.019) and weighted mode (weighted mode: OR 1.1408; 95% CI 1.0072–1.2921; p = 0.0461) confirmed the IBD-rosacea association.

The two main subtypes of IBD: UC and CD, were also analyzed. Cochran's Q test revealed no significant heterogeneity in the analysis of UC on rosacea (Q = 20.8205; *p* = 0.6493). No directional pleiotropy bias was detected (Intercept = − 0.0239; *p* = 0.4728). No SNP outliers were found in the MR-PRESSO global test (RSSobs = 22.9609; *p* = 0.6440) and leave-one-out MR analysis. UC was causally associated with rosacea (IVW: OR 1.2030; 95% CI 1.0867–1.3318; *p* = 0.0004). WM (WM: OR 1.2135; 95% CI 1.0466–1.4070; *p* = 0.0104) confirmed the UC-rosacea association.

Cochran's Q test revealed no significant heterogeneity in the analysis of CD on rosacea (Q = 28.6333; p = 0.6844). No directional pleiotropy bias was detected (Intercept = 0.0027; *p* = 0.8912). No SNP outliers were found in the MR-PRESSO global test (RSSobs = 29.9833; *p* = 0.7020) and leave-one-out analysis. CD was also causally associated with rosacea (IVW: OR 1.1291; 95% CI 1.0444–1.2206; *p* = 0.0023). WM (WM: OR 1.1455; 95% CI 1.0227–1.2830; *p* = 0.0188) confirmed the CD-rosacea association.

### The causal effect of rosacea on IBD and its subtypes

In the reverse-direction MR analysis, significant at the genome-wide level (*p* < 5 × 10^-6^) and independently inherited (*r*^*2*^ < 0.001 and distance > 10,000 kb) from the 16,380,452 SNPs, 13 SNPs were tentatively selected as IVs for rosacea. No SNP associated with IBD-relevant traits was found by inquiring about the PhenoScanner V2 database. One SNP rs2298897 was excluded from the analysis because of being palindromic. Finally, 12 SNPs were accepted for MR analysis evaluating the causative influence of rosacea on IBD, UC, and CD risk.

Based on F statistics (F = 4.63) and the proportion of variance explained (R^2^ = 0.03%), our reverse-direction MR analyses would be affected by weak instrument bias. Supplementary Tables 1–5 provide a summary and full descriptions of the IVs for each exposure.

Cochran's Q test revealed no significant heterogeneity in the analysis of rosacea on total IBD (Q = 6.4603; *p* = 0.1184). In the MR-Egger test, no directional pleiotropy bias was detected (Intercept = − 0.0004; *p* = 0.9761). No SNP outliers were found in the MR-PRESSO global test (RSSobs = 7.2150; *p* = 0.8560) and leave-one-out MR analysis. However, no direct causal effect of rosacea on total IBD was found (IVW: OR 0.9818; 95% CI 0.9390–1.0267; p = 0.4209). The results from the WM (WM: OR 0.9683; 95% CI 0.9112–1.0291; *p* = 0.2999) and MR Egger (MR Egger: OR 0.9828; 95% CI 0.9086–1.0631; *p* = 0.6745) were consistent.

The two main subtypes of IBD: UC and CD, were also analyzed. Cochran's Q test revealed no significant heterogeneity in the analysis of UC on rosacea (Q = 7.9962; *p* = 0.7136). No directional pleiotropy bias was detected (Intercept = 0.0062; *p* = 0.7300). No SNP outliers were found in the MR-PRESSO global test (RSSobs = 9.3873; *p* = 0.7590) and leave-one-out MR analysis. No evidence of a causal effect UC on rosacea was found (IVW: OR 0.9941; 95% CI 0.9392–1.0523; p = 0.8390).WM (WM: OR 0.9714; 95% CI 0.8949–1.0545; *p* = 0.4882) and MR Egger (MR Egger: OR 0.9793; 95% CI 0.8857–1.0828; *p* = 0.6921) confirmed these findings.

Cochran's Q test revealed no significant heterogeneity in the analysis of CD on rosacea (Q = 10.5728; p = 0.4797). No directional pleiotropy bias was detected (Intercept = − 0.0123; *p* = 0.5194). No SNP outliers were found in the MR-PRESSO global test (RSSobs = 12.2924; *p* = 0.5280) and leave-one-out MR analysis. There was no causal association between CD and rosacea (IVW: OR 1.0078; 95% CI 0.9497–1.0694; *p* = 0.7982). The results from the WM (WM: OR 0.9751; 95% CI 0.8971–1.0598; *p* = 0.5525) and MR Egger (MR Egger: OR 1.0373; 95% CI 0.9352–1.1505; *p* = 0.5046) were consistent.

**Figure 2:**
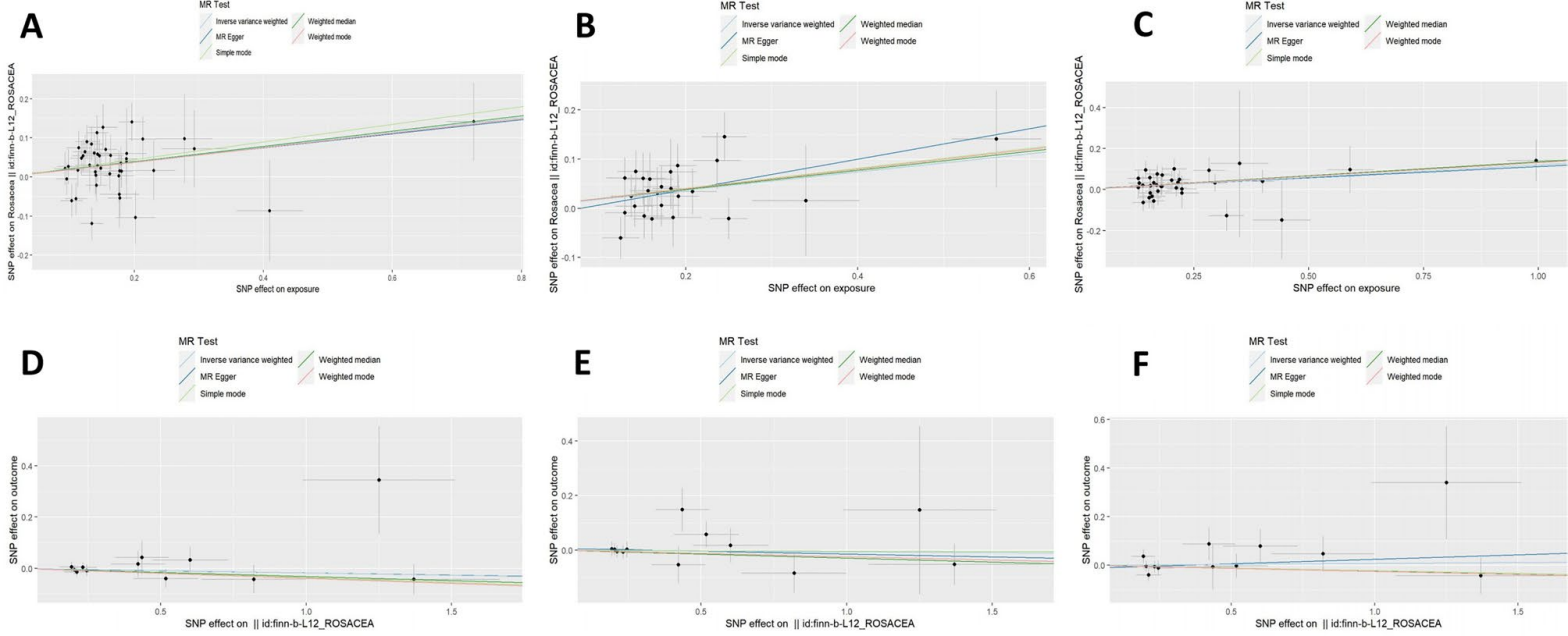
Scatter plots of primary MR analysis. The slope of each line represents the expected MR impact in various models. (**A**) IBD on rosacea; (**B**) UC on rosacea; (**C**) CD on rosacea; (**D**) rosacea on IBD; (**E**) rosacea on UC; (**F**) rosacea on CD. *CD* Crohn's disease, *IBD* inflammatory bowel disease, *MR* Mendelian randomization, *UC* ulcerative colitis.

**Figure 3:**
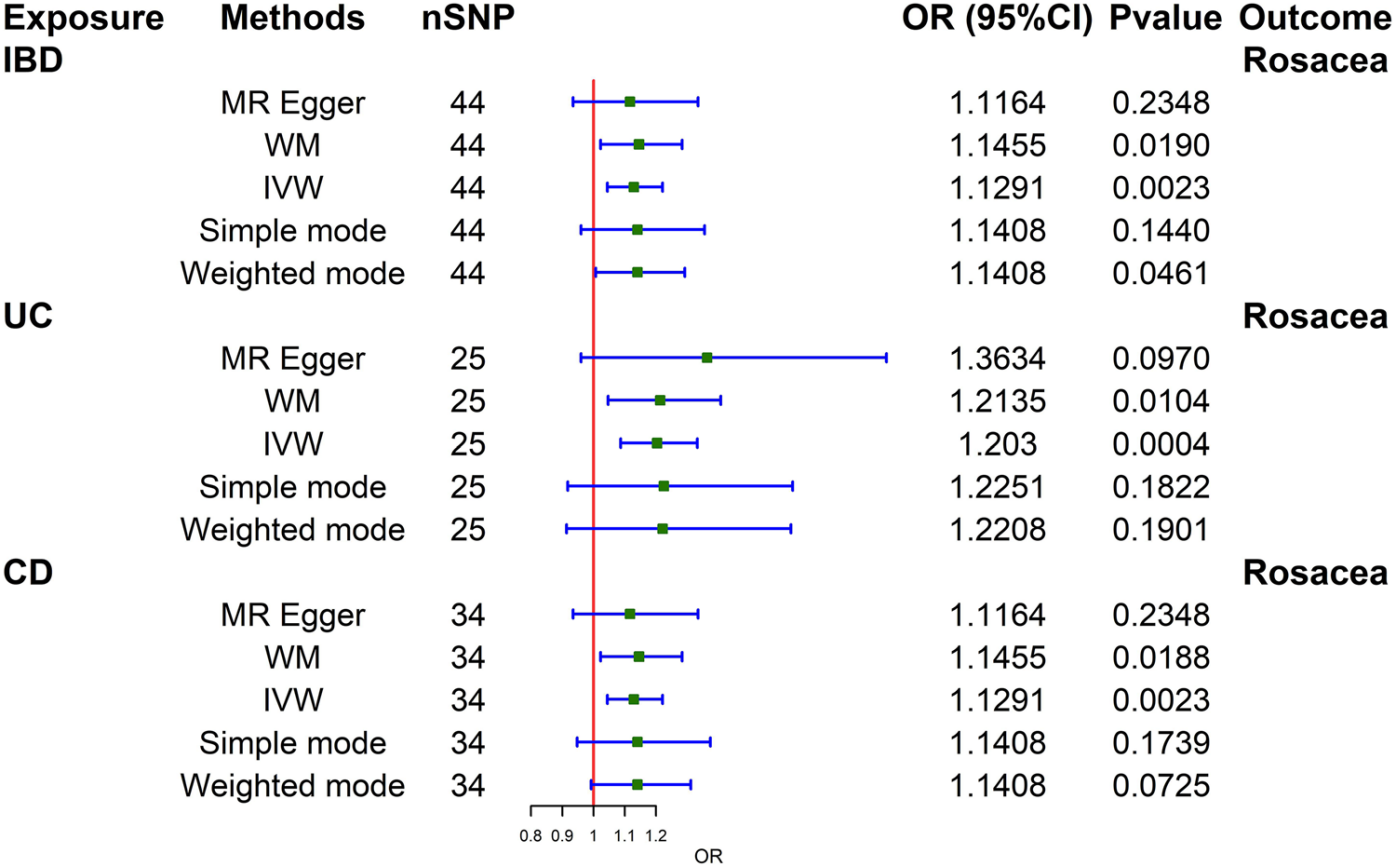
Causal estimates of total IBD, UC, and CD on rosacea given as odds ratios (ORs) with 95% confidence intervals. *CD* Crohn's disease, *CI* confidence intervals, *IBD* inflammatory bowel disease, *IVW* inverse variance-weighted, *OR* odds ratio, *PRESSO* Pleiotropy RESidual Sum and Outlier, *SNP* single-nucleotide polymorphism, *UC* ulcerative colitis.

**Figure 4:**
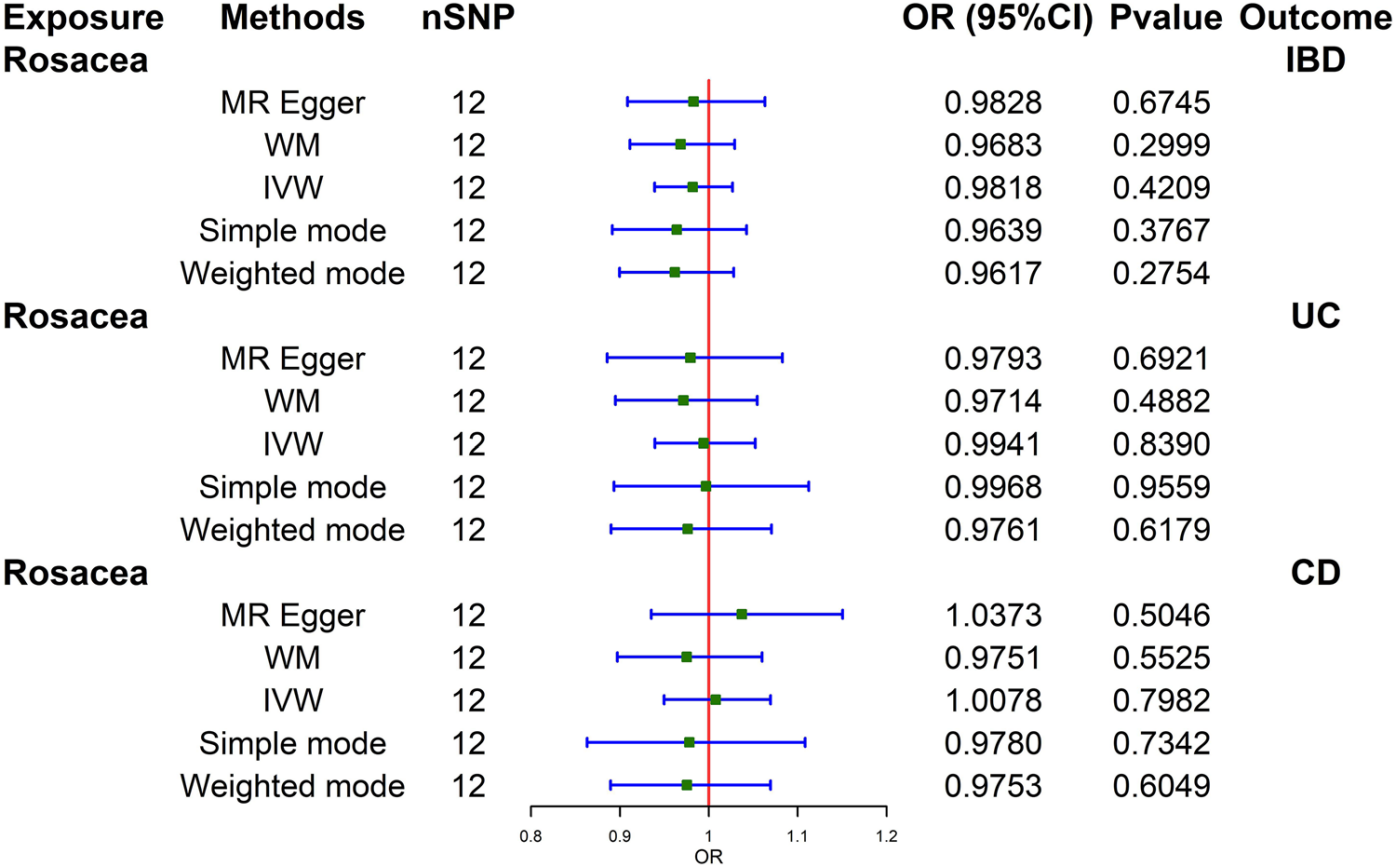
Causal estimates of rosacea on IBD, UC, and CD given as odds ratios (ORs) with 95% confidence intervals. *CD* Crohn's disease, *CI* confidence intervals, *IBD* inflammatory bowel disease, *IVW* inverse variance-weighted, *OR* odds ratio, *PRESSO* Pleiotropy RESidual Sum and Outlier, *SNP* single-nucleotide polymorphism, *UC* ulcerative colitis.

The sensitivity analyses of MR are shown in Table 1. The scatter plots of causal relationships of MR analysis are shown in Fig. 2. The causal relationships are shown in Figs. 3 and 4. The funnel plot, leave-one-out plot, and forest plot of MR analyses are shown in Supplementary Figs. S1–S3.

**Table 1:** Sensitivity analyses of MR.

Exposure	Outcome	Number of Ivs	Heterogeneity test		MR-Egger pleiotropy test		MR-PRESSO results		
			Q	p-value	Intercept	p-value	RSSobs	p-value	Outlier
IBD	Rosacea	44	54.1571	0.1184	0.0018	0.9408	56.5382	0.1400	None
UC	Rosacea	25	20.8205	0.6493	− 0.0239	0.4728	22.9609	0.6440	None
CD	Rosacea	34	28.6333	0.6844	0.0027	0.8912	29.9833	0.7020	None
Rosacea	IBD	12	6.4603	0.8410	− 0.0004	0.9761	7.2150	0.8560	None
Rosacea	UC	12	7.9962	0.7136	0.0062	0.7300	9.3873	0.7590	None
Rosacea	CD	12	10.5728	0.4797	− 0.0123	0.5194	12.2924	0.5280	None

*CD* Crohn’s disease, *IBD* inflammatory bowel disease, *MR* Mendelian randomization, *UC* ulcerative colitis.

## Discussion

We employed MR analysis to investigate the bidirectional association between IBD and rosacea in this investigation. We found evidence that genetic predisposition to IBD, UC, and CD was linked to an elevated risk of rosacea. Besides, we did not find any evidence that the genetic predisposition to rosacea was linked to an elevated risk of IBD, UC, or CD.

Previous observational studies have evaluated the possible link between IBD and rosacea. Contradictory results were, however, reported. Spoendlin et al. carried out a case–control analysis on the association between IBD and rosacea, stratified by IBD disease duration and severity, they provided evidence that patients with UC and CD may be at increased risk of rosacea^42^. Egeberg et al. performed a nationwide cohort study including a total of 49,475 patients with rosacea and 4,312,213 general population controls, they found that the prevalence of CD and UC was higher among patients with rosacea when compared to the control subjects^41^. Li et al. did a prospective study, they identified 149 cases of CD and 215 cases of UC during 1,856,587 person-years (1991–2011), and they found that rosacea was not associated with risk of UC but was significantly associated with an increased risk of CD^10^. Kim et al*.* conducted a study to examine the association between inflammatory bowel disease (IBD) and the risk of rosacea in a cohort of patients, who were carefully matched with control subjects based on age and sex. The findings revealed that male patients with IBD displayed a significantly higher risk of developing rosacea compared to their female counterparts. Furthermore, individuals below the age of 30 exhibited an increased susceptibility to rosacea^11^. A meta-analysis of 13 separate studies involving 5,051,356 participants conducted by Jing Han et al. concluded that the relative risks (RR) of IBD in rosacea patients rosacea were 1.32 (IBD, 95% CI 1.18–1.49), 1.19 (UC, 95% CI 1.02–1.38), and 1.52 (CD, 95% CI 1.25–1.84) respectively, and the RR (95% CI) of rosacea in IBD patients were 1.66 (IBD 1.50–1.84), 1.69 (UC 1.48–1.93) and 2.08 (CD 1.26–3.46) respectively (P < 0.05)^8^. Another meta-analysis by Wang et al. consisting of three case–control and three cohort studies, concluded that the meta-analysis of case–control studies found a marginally increased risk of CD (OR 1.30; 95% CI 0.99–1.69) and a considerably increased risk of UC (OR 1.64; 95% CI 1.43–1.89) in rosacea patients, and the meta-analysis of cohort studies found a considerably increased odds of CD (hazard ratio (HR) 1.58; 95% CI 1.14–2.20) and UC (HR 1.18, 95% CI 1.01–1.37) in rosacea patients^25^. A recent meta-analysis indicates a bidirectional association between IBD and rosacea^43^. These reported associations and our findings differ to some extent. We assume that the difference is due to the two distinct analytical methods themselves. Unavoidable clinical confounding variables may alter exposure and outcome, reducing the capacity to make reliable causal inferences in observational research. As a result, even if observational studies discovered a strong association, it was unable to identify a direct causal connection. By using genetic instrumental variables, Mendelian randomization can avoid the impact of these confounding factors and produce a reasonably accurate causal evaluation.

Our findings of MR analysis provided evidence for a causal impact of IBD, UC, and CD on rosacea, but not vice versa. Additionally, our subanalyses verified that patients with UC and CD have a higher risk of developing rosacea. These findings can be explained by two potential explanations. Firstly, it is plausible that individuals with inflammatory bowel disease (IBD) have a higher susceptibility to developing rosacea, while rosacea itself does not contribute to the development of IBD. Secondly, the limited number of rosacea cases in the Fingen database may introduce bias in the analysis of reverse causality due to insufficient sample size. We propose that inflammation associated with IBD could be one of the causative factors for rosacea. Additionally, certain medications used in the treatment of IBD, such as topical or systemic corticosteroids, have been reported in the literature to be associated with the onset of rosacea^44^. It is worth noting that symptoms of medication-induced rosacea often resolve after discontinuation of the triggering medication. Furthermore, although ulcerative colitis (UC) and Crohn’s disease (CD) both fall under the umbrella of IBD, they have distinct clinical presentations and pathological characteristics. CD is typically characterized by weight loss, while bloody stool is a hallmark of UC. UC primarily affects the colon and rectum, whereas CD can involve the entire gastrointestinal tract. The difference in risk between these two diseases may be attributed to their inherent distinctions.

The underlying mechanisms of the link between IBD and rosacea have not yet been fully elucidated. IBD and rosacea are both chronic inflammatory conditions of the skin and gastrointestinal organs involving the interplay between genetic and immunological elements, this raises the possibility that they are related in some way. IBD and rosacea both have abnormalities in innate and adaptive immunity. and they share some common risk factors such as smoking status^23,41^, obesity^24,30^, and small intestinal bacterial overgrowth^45,46^. A systematic review and meta-analysis have revealed a potential association specifically with phymatous rosacea, which is a subtype of rosacea^20^. Li et al. found significant associations between past and current smoking and rosacea^19^. Prospective studies found that alcohol usage was linked to a higher risk of IBD relapse, although evidence linking the consumption of alcoholic beverages and the development of new-onset IBD was controversial^18^. Karban et al. found that never smoking and formerly smoking increase the risk of UC, whereas smoking exacerbates the course of CD. A study has revealed a potential association between IBD and BMI^30^ while another twin study suggests a possible association between rosacea and BMI^24^.

We would want to highlight some of our study's merits while simultaneously addressing some of its limitations. The main merit of our work is that, to the greatest of our knowledge, we employed a 2-sample MR technique for the first time to explore the bidirectional relationship between rosacea and IBD. In comparison to observational studies, this strategy is less susceptible to confounding factors, reverse causation, and exposures that do not differ across groups. Furthermore, the subtypes of inflammatory bowel disease are strictly defined to eliminate the effect of disease coexistence on outcomes. Third, a sensitivity analysis was carried out to make sure that causal estimates were consistent and that the results were reliable. There are certain limits as well. Because IVs were few, a lower *p*(*p* < 10^–6^) threshold was defined when rosacea was used as an instrumental variable for exposure. Moreover, the F-statistic test indicated that the instrument was weak. Thus, the negative result of our reverse-direction MR analyses should be interpreted with caution. Second, individuals of other ethnicities cannot be confidently predicted based on the current research because it was conducted on individuals of European ancestry. Finally, although we matched every chosen SNP to the PhenoScanner database to identify potential confounding variables and related horizontal polymorphisms, this technique does not eradicate the influence of horizontal polymorphisms because many genetic variations' specific biological function is unclear. In two-sample MR analysis, substantial differences in population characteristics, such as age, sex, socio-economic background, and more, may exist between the two samples. These differences can not only impact the interpretation of causal estimates but also compromise the validity of causal inferences. However, exclusively selecting FinnGen for both rosacea and IBD poses certain difficulties and disadvantages. Firstly, it could be challenging to find suitable samples for both diseases. Secondly, this approach may be categorized as a one-sample MR analysis, resulting in lower statistical power compared to a two-sample MR analysis. Currently, most two-sample Mendelian randomization studies aim to leverage data from different databases to enhance the study’s statistical power. Future studies will partially address these limitations once higher-quality GWAS research becomes available.

## Conclusions

Our findings of MR analysis provided evidence for a causal impact of IBD, UC, and CD on rosacea, but not vice versa. The primary IBD subtypes UC and CD appear to have a causal association with rosacea as well.

The elevated incidence of rosacea in patients with IBD should be recognized by doctors to make an early diagnosis and initiate specialized therapy. To further understand the pathophysiological processes behind this association, more research is required.

### Supplementary Information

Supplementary Information.

## Data Availability

All data that supporting the findings of this study were available in the public domain through FinnGen and IIBDGC Consortium.
