# Supplementary material for: Detecting potential causal relationship between inflammatory bowel disease and rosacea using bi-directional Mendelian randomization
Source: Sci Rep. 2023 Sep 9;13:14910. doi: 10.1038/s41598-023-42073-6 (PMC10492847; doi:10.1038/s41598-023-42073-6)
Supplement: Supplementary file 1 — Supplementary Information. [file 41598_2023_42073_MOESM1_ESM.docx]

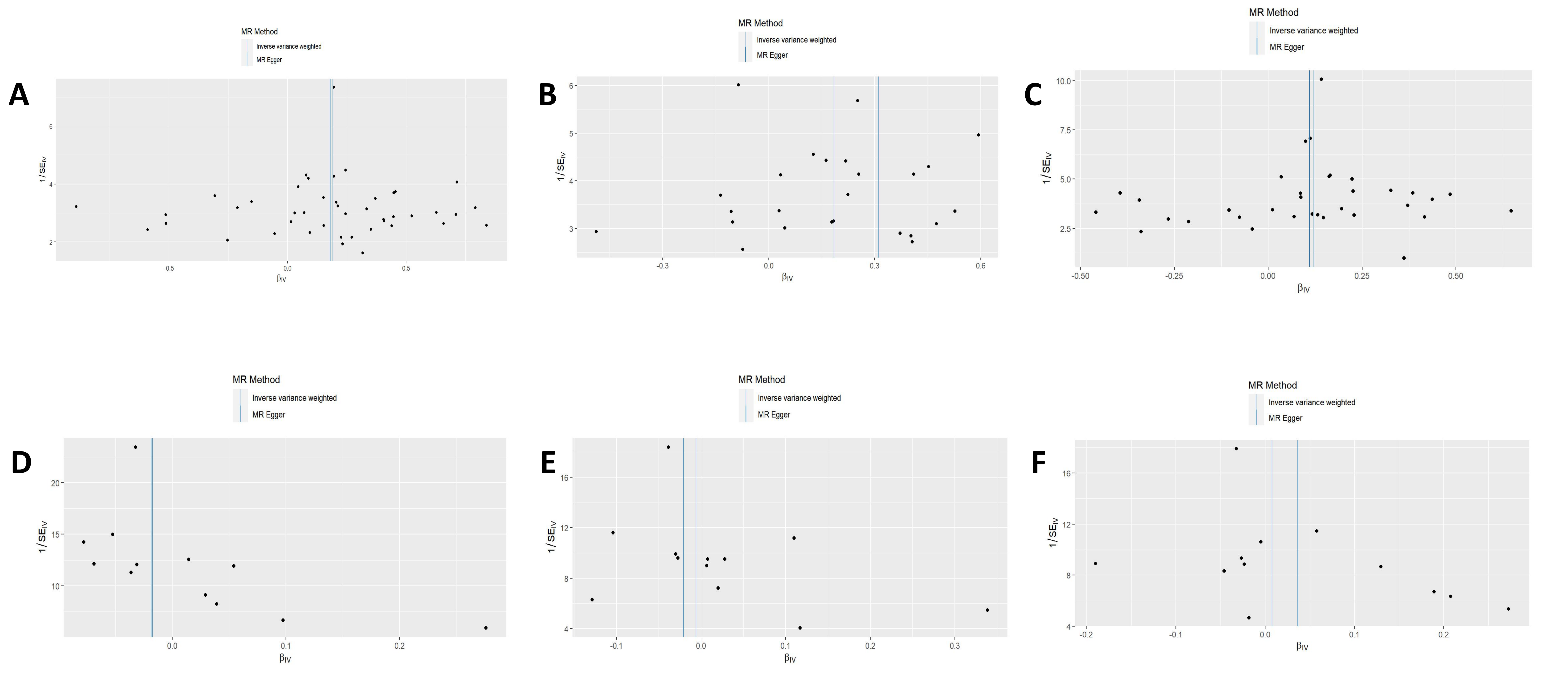


**Figure S1** Funnel plot of MR analyses. (A) IBD on rosacea; (B) UC on rosacea; (C) CD on rosacea; (D) rosacea on IBD; (E) rosacea on UC; (F) rosacea on CD. CD, Crohn's disease; IBD, inflammatory bowel disease; MR, Mendelian randomization; UC, ulcerative colitis.


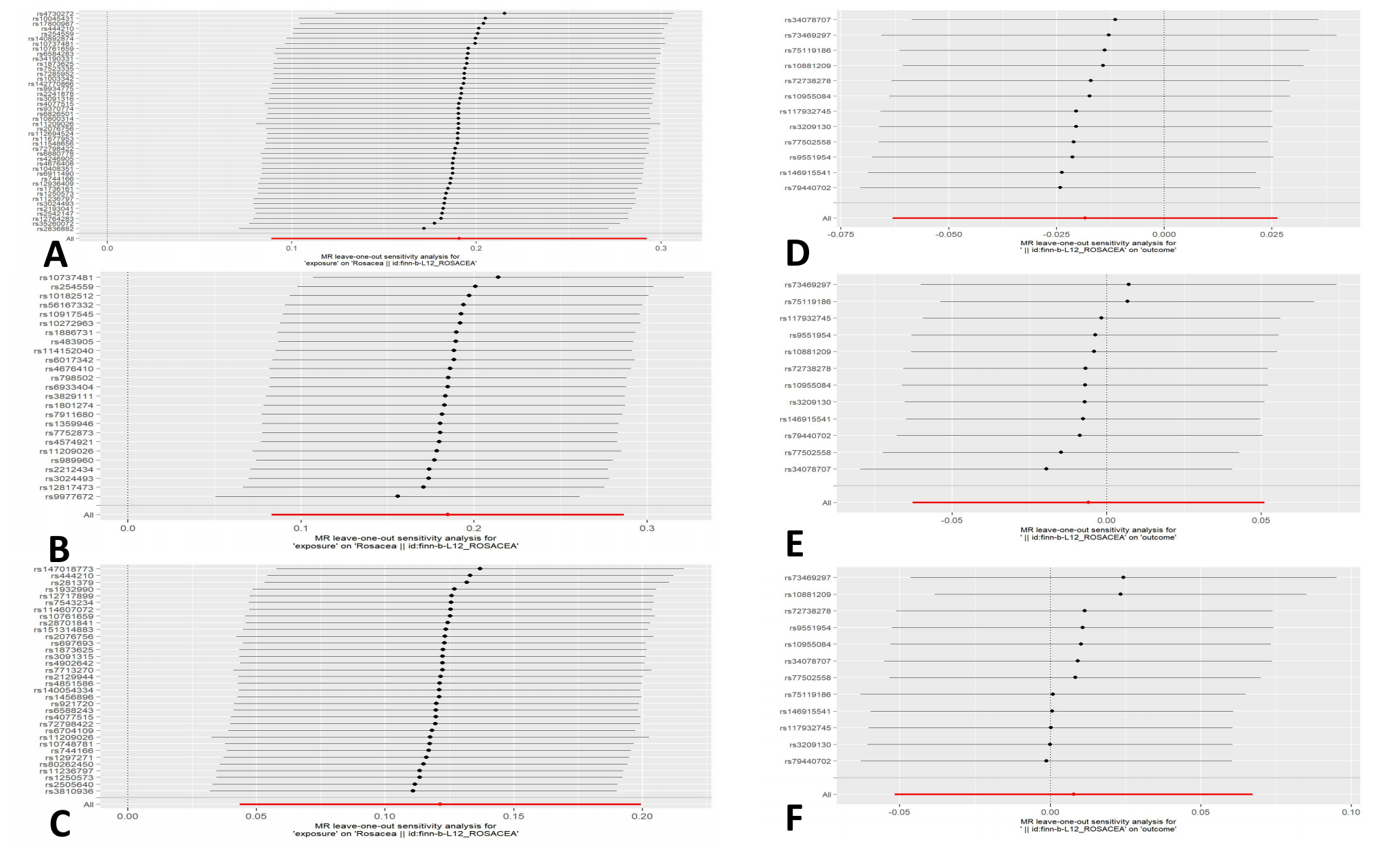


**Figure S2** Leave-one-out plot of MR analyses. (A) IBD on rosacea; (B) UC on rosacea; (C) CD on rosacea; (D) rosacea on IBD; (E) rosacea on UC; (F) rosacea on CD. CD, Crohn's disease; IBD, inflammatory bowel disease; MR, Mendelian randomization; UC, ulcerative colitis.


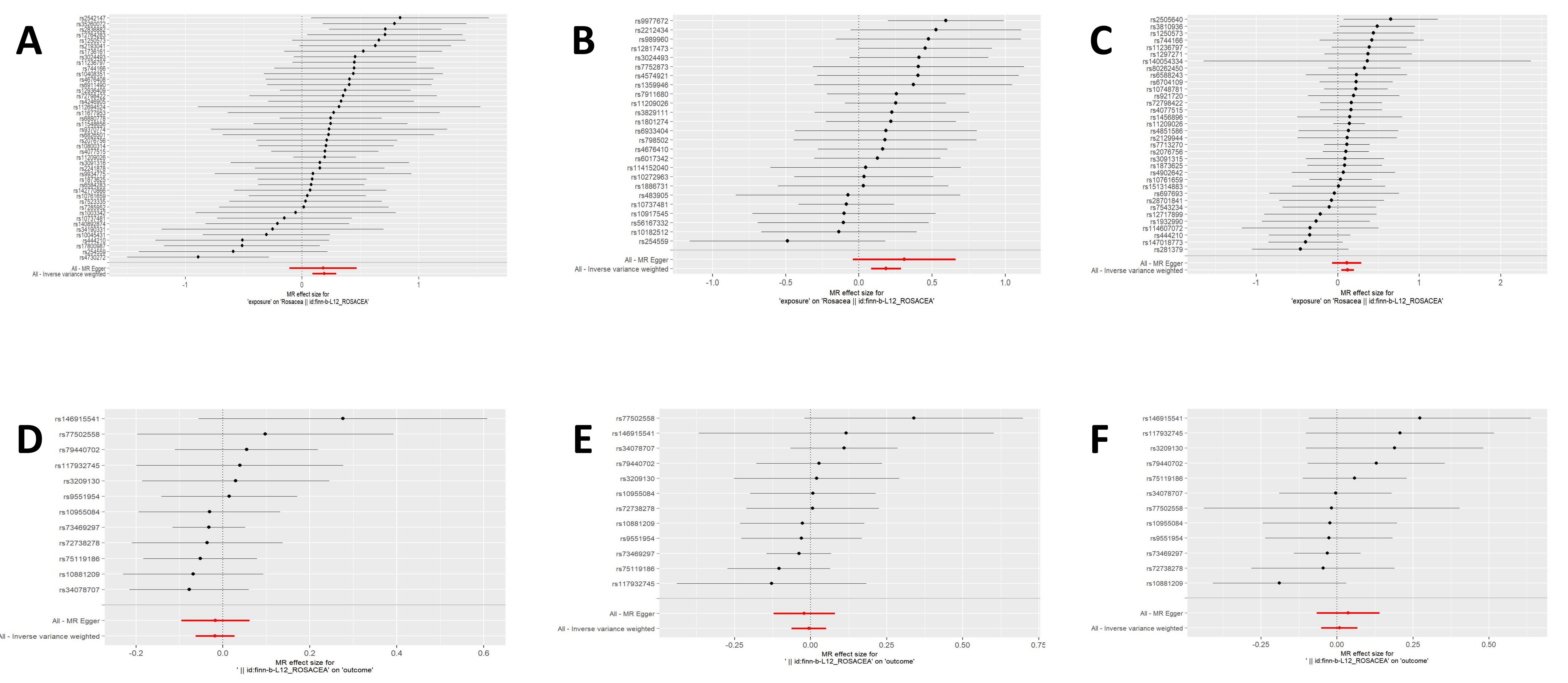


**Figure S3** Forrest plot MR analyses. (A) IBD on rosacea; (B) UC on rosacea; (C) CD on rosacea; (D) rosacea on IBD; (E) rosacea on UC; (F) rosacea on CD. CD, Crohn's disease; IBD, inflammatory bowel disease; MR, Mendelian randomization; UC, ulcerative colitis.

| **Table S1** Summary of Genetic Instruments identified for MR Analyses | | | | |
| --- | --- | --- | --- | --- |
| **Exposure** | **Significant level** | **No. of SNPs** | **F statistics**** | **Explained variance (R**2) * |
| **IBD** | | | | |
| IBD | 5.00E-08 | 44 | 22.99 | 1.20% |
| UC | 5.00E-08 | 25 | 22.12 | 0.85% |
| CD | 5.00E-08 | 34 | 22.59 | 1.22% |
| **Rosacea** | 1.00E-06 | 12 | 4.63 | 0.03% |
| *Variation explained(R2) by the included SNPs was computed using the formula: R2 = 2 × (1 – MAF) × MAF × β2/(SE2 × N) where SE (β) is the SE (β coefficient) for effect size, MAF is the minimum allele frequency for each SNP, and N is the sample size. **F-statistic to evaluate the total strength of the selected SNPs was estimated using the formula [(N – k – 1)/k] × [R2/(1 – R2)] ,where N is the sample size, k is the total number of SNPs selected for MR analysis.IBD: Inflammatory Bowel Disease; CD: Crohn’s Disease; UC: Ulcerative Colitis. | | | | |

| **Table S2** Instrumental variables for IBD (significant level of p < 1e-08) | | | | | | | | |
| --- | --- | --- | --- | --- | --- | --- | --- | --- |
| **SNP** | **EA** | **non-EA** | **Beta** | **se** | **pval.exposure** | **palindromic** | **Rosacea** | |
|  |  |  |  |  |  |  | Beta | se |
| rs1003342 | A | G | 0.095001042 | 0.0168 | 1.67E-08 | FALSE | -0.0052 | 0.0416 |
| rs10045431 | A | C | -0.177405661 | 0.0189 | 6.59E-21 | FALSE | 0.0543 | 0.0493 |
| rs10408351 | A | G | 0.137803506 | 0.0221 | 4.23E-10 | FALSE | 0.0605 | 0.0539 |
| rs10737481 | T | G | -0.141102842 | 0.017 | 1.19E-16 | FALSE | 0.0214 | 0.0416 |
| rs10761659 | A | G | -0.161895596 | 0.0172 | 4.07E-21 | FALSE | -0.0073 | 0.0415 |
| rs10800314 | A | C | -0.143096999 | 0.0179 | 1.17E-15 | FALSE | -0.0294 | 0.0424 |
| rs11209026 | A | G | -0.726290407 | 0.0422 | 1.76E-66 | FALSE | -0.1411 | 0.0989 |
| rs11236797 | A | C | 0.155703761 | 0.017 | 4.75E-20 | FALSE | 0.0698 | 0.0422 |
| rs112694524 | A | G | 0.18830363 | 0.0303 | 5.39E-10 | FALSE | 0.0598 | 0.1163 |
| rs11548656 | A | G | 0.292796475 | 0.0507 | 7.72E-09 | FALSE | 0.0716 | 0.0985 |
| rs11677953 | A | G | 0.09759847 | 0.0171 | 1.05E-08 | FALSE | 0.0265 | 0.0452 |
| rs1250573 | A | G | -0.113605525 | 0.019 | 2.21E-09 | FALSE | -0.0748 | 0.0431 |
| rs12764283 | A | G | 0.126597409 | 0.0179 | 1.57E-12 | FALSE | 0.0899 | 0.043 |
| rs12936409 | T | C | 0.145700796 | 0.0168 | 3.87E-18 | FALSE | 0.0539 | 0.0416 |
| rs140892874 | T | C | -0.409593623 | 0.0512 | 1.28E-15 | FALSE | 0.0864 | 0.1288 |
| rs142770866 | A | G | 0.229999995 | 0.0337 | 8.14E-12 | FALSE | 0.0161 | 0.0765 |
| rs1736161 | A | G | -0.123298218 | 0.0174 | 1.34E-12 | FALSE | -0.0646 | 0.0425 |
| rs17800987 | A | G | -0.201700116 | 0.0305 | 3.71E-11 | FALSE | 0.1037 | 0.0687 |
| rs1873625 | A | C | 0.177300642 | 0.0179 | 3.71E-23 | FALSE | 0.0155 | 0.0422 |
| rs2076756 | A | G | -0.187595441 | 0.0186 | 5.59E-24 | FALSE | -0.0398 | 0.0578 |
| rs2193041 | A | G | -0.133702837 | 0.0172 | 6.91E-15 | FALSE | -0.0839 | 0.0443 |
| rs2241878 | T | C | -0.148001294 | 0.0169 | 1.75E-18 | FALSE | -0.0224 | 0.0419 |
| rs2542147 | T | G | -0.151299749 | 0.0227 | 2.79E-11 | FALSE | -0.127 | 0.0588 |
| rs254559 | A | C | 0.102800242 | 0.0172 | 2.08E-09 | FALSE | -0.0607 | 0.0424 |
| rs2836882 | A | G | -0.19629473 | 0.0201 | 1.49E-22 | FALSE | -0.1402 | 0.0483 |
| rs3024493 | A | C | 0.212996243 | 0.0222 | 8.48E-22 | FALSE | 0.097 | 0.0571 |
| rs3091316 | A | G | -0.112497032 | 0.0191 | 3.59E-09 | FALSE | -0.0172 | 0.0439 |
| rs34190331 | A | G | 0.176898547 | 0.0303 | 5.39E-09 | FALSE | -0.0447 | 0.0859 |
| rs35260072 | A | C | -0.142197407 | 0.017 | 7.07E-17 | FALSE | -0.1127 | 0.0447 |
| rs4077515 | T | C | 0.17940063 | 0.0172 | 1.50E-25 | FALSE | 0.0351 | 0.042 |
| rs4246905 | T | C | -0.163001401 | 0.0197 | 1.42E-16 | FALSE | -0.0544 | 0.0519 |
| rs444210 | A | G | -0.109502416 | 0.0168 | 7.39E-11 | FALSE | 0.0561 | 0.0416 |
| rs4676408 | A | G | 0.118102986 | 0.0181 | 6.62E-11 | FALSE | 0.0481 | 0.0433 |
| rs4730272 | A | G | 0.134102481 | 0.0178 | 4.50E-14 | FALSE | -0.1195 | 0.0416 |
| rs6584283 | T | C | 0.180302856 | 0.0169 | 1.70E-26 | FALSE | 0.0141 | 0.0418 |
| rs6826501 | T | C | -0.092795346 | 0.0169 | 4.12E-08 | FALSE | -0.021 | 0.0429 |
| rs6880778 | A | G | -0.187800541 | 0.0173 | 2.14E-27 | FALSE | -0.046 | 0.0419 |
| rs6911490 | T | C | 0.1428008 | 0.0208 | 6.82E-12 | FALSE | 0.0579 | 0.0514 |
| rs72798422 | T | C | -0.27759974 | 0.0431 | 1.19E-10 | FALSE | -0.0976 | 0.1139 |
| rs7285952 | T | G | 0.17600163 | 0.0235 | 7.60E-14 | FALSE | 0.0026 | 0.0652 |
| rs744166 | A | G | 0.120703213 | 0.0172 | 2.16E-12 | FALSE | 0.0539 | 0.0421 |
| rs7523335 | A | G | -0.140504219 | 0.0225 | 4.16E-10 | FALSE | -0.0042 | 0.0468 |
| rs9370774 | T | C | 0.13070365 | 0.0219 | 2.54E-09 | FALSE | 0.0303 | 0.0675 |
| rs9934775 | T | C | -0.139595458 | 0.0232 | 1.72E-09 | FALSE | -0.0131 | 0.06 |

| **Table S3** Instrumental variables for UC (significant level of p < 1e-08) | | | | | | | | |
| --- | --- | --- | --- | --- | --- | --- | --- | --- |
| **SNP** | **EA** | **non-EA** | **Beta** | **se** | **pval.exposure** | **palindromic** | **Rosacea** | |
|  |  |  |  |  |  |  | **Beta** | **se** |
| rs10182512 | A | G | 0.160799953 | 0.0223 | 5.19E-13 | FALSE | -0.0219 | 0.0435 |
| rs10272963 | T | C | -0.171904008 | 0.0216 | 1.69E-15 | FALSE | -0.0058 | 0.0417 |
| rs10737481 | T | G | -0.250103733 | 0.0216 | 4.37E-31 | FALSE | 0.0214 | 0.0416 |
| rs10917545 | A | G | -0.185101417 | 0.0335 | 3.29E-08 | FALSE | 0.0189 | 0.0591 |
| rs11209026 | A | G | -0.561697954 | 0.0517 | 1.58E-27 | FALSE | -0.1411 | 0.0989 |
| rs114152040 | A | G | 0.339603045 | 0.0623 | 4.95E-08 | FALSE | 0.0154 | 0.1127 |
| rs12817473 | A | G | -0.190700563 | 0.0217 | 1.71E-18 | FALSE | -0.0863 | 0.0444 |
| rs1359946 | A | G | 0.158302051 | 0.0269 | 3.84E-09 | FALSE | 0.0588 | 0.0546 |
| rs1801274 | A | G | 0.182896392 | 0.0217 | 3.78E-17 | FALSE | 0.0399 | 0.0414 |
| rs1886731 | T | C | 0.1405008 | 0.0221 | 2.25E-10 | FALSE | 0.0041 | 0.0417 |
| rs2212434 | T | C | 0.141898788 | 0.0213 | 2.46E-11 | FALSE | 0.0748 | 0.0422 |
| rs254559 | A | C | 0.12430395 | 0.0215 | 7.63E-09 | FALSE | -0.0607 | 0.0424 |
| rs3024493 | A | C | 0.236296669 | 0.0276 | 1.09E-17 | FALSE | 0.097 | 0.0571 |
| rs3829111 | A | G | 0.156302648 | 0.0214 | 2.89E-13 | FALSE | 0.0351 | 0.0421 |
| rs4574921 | T | C | 0.150598665 | 0.0256 | 4.24E-09 | FALSE | 0.0607 | 0.0529 |
| rs4676410 | A | G | 0.207802476 | 0.0284 | 2.46E-13 | FALSE | 0.0337 | 0.0469 |
| rs483905 | A | G | 0.128903199 | 0.0228 | 1.57E-08 | FALSE | -0.0095 | 0.0504 |
| rs56167332 | A | C | 0.151595991 | 0.0231 | 5.30E-11 | FALSE | -0.0161 | 0.0452 |
| rs6017342 | A | C | -0.191305794 | 0.024 | 1.39E-15 | FALSE | -0.0242 | 0.042 |
| rs6933404 | T | C | -0.166798663 | 0.0252 | 3.69E-11 | FALSE | -0.0307 | 0.0529 |
| rs7752873 | T | C | 0.182296556 | 0.0303 | 1.83E-09 | FALSE | 0.0741 | 0.067 |
| rs7911680 | A | C | 0.171799729 | 0.0213 | 8.27E-16 | FALSE | 0.0438 | 0.0415 |
| rs798502 | A | C | 0.136495745 | 0.0239 | 1.21E-08 | FALSE | 0.0244 | 0.0435 |
| rs989960 | T | C | -0.129095531 | 0.0215 | 1.77E-09 | FALSE | -0.0613 | 0.0416 |
| rs9977672 | A | G | -0.245005798 | 0.0261 | 6.21E-21 | FALSE | -0.1455 | 0.0494 |

| **Table S4** Instrumental variables for CD (significant level of p < 1e-08) | | | | | | | | |
| --- | --- | --- | --- | --- | --- | --- | --- | --- |
| **SNP** | **EA** | **non-EA** | **Beta** | **se** | **pval.exposure** | **palindromic** | **Rosacea** | |
|  |  |  |  |  |  |  | **Beta** | **se** |
| rs10182512 | A | G | 0.160799953 | 0.0223 | 5.19E-13 | FALSE | -0.0219 | 0.0435 |
| rs10272963 | T | C | -0.171904008 | 0.0216 | 1.69E-15 | FALSE | -0.0058 | 0.0417 |
| rs10737481 | T | G | -0.250103733 | 0.0216 | 4.37E-31 | FALSE | 0.0214 | 0.0416 |
| rs10917545 | A | G | -0.185101417 | 0.0335 | 3.29E-08 | FALSE | 0.0189 | 0.0591 |
| rs11209026 | A | G | -0.561697954 | 0.0517 | 1.58E-27 | FALSE | -0.1411 | 0.0989 |
| rs114152040 | A | G | 0.339603045 | 0.0623 | 4.95E-08 | FALSE | 0.0154 | 0.1127 |
| rs12817473 | A | G | -0.190700563 | 0.0217 | 1.71E-18 | FALSE | -0.0863 | 0.0444 |
| rs1359946 | A | G | 0.158302051 | 0.0269 | 3.84E-09 | FALSE | 0.0588 | 0.0546 |
| rs1801274 | A | G | 0.182896392 | 0.0217 | 3.78E-17 | FALSE | 0.0399 | 0.0414 |
| rs1886731 | T | C | 0.1405008 | 0.0221 | 2.25E-10 | FALSE | 0.0041 | 0.0417 |
| rs2212434 | T | C | 0.141898788 | 0.0213 | 2.46E-11 | FALSE | 0.0748 | 0.0422 |
| rs254559 | A | C | 0.12430395 | 0.0215 | 7.63E-09 | FALSE | -0.0607 | 0.0424 |
| rs3024493 | A | C | 0.236296669 | 0.0276 | 1.09E-17 | FALSE | 0.097 | 0.0571 |
| rs3829111 | A | G | 0.156302648 | 0.0214 | 2.89E-13 | FALSE | 0.0351 | 0.0421 |
| rs4574921 | T | C | 0.150598665 | 0.0256 | 4.24E-09 | FALSE | 0.0607 | 0.0529 |
| rs4676410 | A | G | 0.207802476 | 0.0284 | 2.46E-13 | FALSE | 0.0337 | 0.0469 |
| rs483905 | A | G | 0.128903199 | 0.0228 | 1.57E-08 | FALSE | -0.0095 | 0.0504 |
| rs56167332 | A | C | 0.151595991 | 0.0231 | 5.30E-11 | FALSE | -0.0161 | 0.0452 |
| rs6017342 | A | C | -0.191305794 | 0.024 | 1.39E-15 | FALSE | -0.0242 | 0.042 |
| rs6933404 | T | C | -0.166798663 | 0.0252 | 3.69E-11 | FALSE | -0.0307 | 0.0529 |
| rs7752873 | T | C | 0.182296556 | 0.0303 | 1.83E-09 | FALSE | 0.0741 | 0.067 |
| rs7911680 | A | C | 0.171799729 | 0.0213 | 8.27E-16 | FALSE | 0.0438 | 0.0415 |
| rs798502 | A | C | 0.136495745 | 0.0239 | 1.21E-08 | FALSE | 0.0244 | 0.0435 |
| rs989960 | T | C | -0.129095531 | 0.0215 | 1.77E-09 | FALSE | -0.0613 | 0.0416 |
| rs9977672 | A | G | -0.245005798 | 0.0261 | 6.21E-21 | FALSE | -0.1455 | 0.0494 |

| **Table S5** Instrumental variables for rosacea (significant level of p < 1e-06) | | | | | | | | | | | |
| --- | --- | --- | --- | --- | --- | --- | --- | --- | --- | --- | --- |
| **SNP** | **EA/non-EA** | **Beta** | **se** | **pval.exposure** | **palindromic** | **IBD** | | **UC** | | **CD** | |
|  |  |  |  |  |  | **Beta** | **se** | **Beta** | **se** | **Beta** | **se** |
| rs10881209 | C/T | -0.2124 | 0.0438 | 1.23E-06 | FALSE | 0.0146 | 0.0175 | 0.0058 | 0.0221 | 0.0403 | 0.0238 |
| rs10955084 | T/C | 0.2037 | 0.0421 | 1.29E-06 | FALSE | -0.0063 | 0.0169 | 0.0016 | 0.0214 | -0.0047 | 0.0230 |
| rs117932745 | A/G | 0.4228 | 0.0921 | 4.44E-06 | FALSE | 0.0166 | 0.0513 | -0.0544 | 0.0672 | 0.0878 | 0.0668 |
| rs146915541 | A/G | 1.2502 | 0.2617 | 1.79E-06 | FALSE | 0.3450 | 0.2117 | 0.1462 | 0.3091 | 0.3408 | 0.2331 |
| rs3209130 | G/A | 0.1941 | 0.0418 | 3.38E-06 | FALSE | 0.0057 | 0.0213 | 0.0039 | 0.0269 | 0.0367 | 0.0289 |
| rs34078707 | G/T | 0.5186 | 0.1112 | 3.11E-06 | FALSE | -0.0403 | 0.0364 | 0.0570 | 0.0464 | -0.0024 | 0.0489 |
| rs72738278 | C/T | -0.2466 | 0.0534 | 3.89E-06 | FALSE | 0.0089 | 0.0218 | -0.0017 | 0.0274 | 0.0113 | 0.0296 |
| rs73469297 | T/C | 1.3709 | 0.296 | 3.64E-06 | FALSE | -0.0440 | 0.0585 | -0.0528 | 0.0746 | -0.0440 | 0.0766 |
| rs75119186 | A/G | 0.8199 | 0.1791 | 4.68E-06 | FALSE | -0.0428 | 0.0547 | -0.0853 | 0.0706 | 0.0474 | 0.0716 |
| rs77502558 | T/C | 0.4357 | 0.0918 | 2.09E-06 | FALSE | 0.0425 | 0.0655 | 0.1476 | 0.0797 | -0.0078 | 0.0936 |
| rs79440702 | T/C | 0.6019 | 0.1299 | 3.58E-06 | FALSE | 0.0326 | 0.0505 | 0.0168 | 0.0633 | 0.0780 | 0.0693 |
| rs9551954 | C/T | 0.2333 | 0.0483 | 1.39E-06 | FALSE | 0.0034 | 0.0186 | -0.0070 | 0.0235 | -0.0062 | 0.0250 |
